# Chemoresistance in cervical cancer: identifying and exploiting non-coding RNAs

**DOI:** 10.3389/fonc.2026.1913768

**Published:** 2026-10-06

**Authors:** Lilian Makgoo, Zukile Mbita

**Affiliations:** Department of Biochemistry, Microbiology and Biotechnology, University of Limpopo, Polokwane, South Africa

**Keywords:** cervical cancer, circRNAs, drug resistance, lncRNAs, miRNAs, non-coding RNAs, chemoresistance

## Abstract

Drug resistance continues to pose a significant challenge, accounting for a large proportion of relapses and playing a major role in cervical cancer-related mortality worldwide. Emerging evidence has indicated that the aberrant expression of non-coding RNAs, especially circular RNAs (circRNAs), long non-coding RNAs (lncRNAs), and microRNAs (miRNAs) is responsible for drug resistance, which poses a significant challenge to the success of chemotherapy. Addressing drug resistance is crucial for improving patient outcomes and reducing the burden of cervical cancer on healthcare systems. In this review, we summarize the molecular and cellular mechanisms through which circRNAs, miRNAs and lncRNAs mediate drug resistance to common drugs used in the treatment of cervical cancer. This work underscores non-coding RNAs as a promising therapeutic target, offering a potential avenue for overcoming chemoresistance in cervical cancer.

## 1 Introduction

Cervical cancer remains a thorn because in 2022, around 350,000 women succumbed to this disease and approximately 660,000 new cases were reported (1). Despite the considerable progress made in the fight against cervical cancer, vaccination and screening coverage remains incomplete, leading to the continued prevalence of this disease among women across the globe (2). The treatment of cervical cancer is influenced by several factors, including the stage of cancer, the patient’s overall health, and the extent to which the cancer has metastasized. Treatment modalities available for cervical cancer include surgery, radiation, chemotherapy, and immunotherapy (3, 4). Despite substantial progress in both conventional and targeted therapy, treatment failure continues to be the main hurdle in treating cervical cancer (5). Some patients may develop resistance to treatments either from the beginning (intrinsic) or following an initial positive response to therapy (acquired). This resistance diminishes therapeutic effectiveness and leads to unfavorable clinical results (5).

Chemoresistance continues to be a major barrier to effective treatment of cervical cancer, especially in advanced and recurrent cases. Chemoresistance in cervical cancer can be achieved by various intricate mechanisms, such as drug efflux mediated by ATP-binding cassette (ABC) transporter (6, 7), altering the expression of proteins that are targeted by anticancer drugs (8), drug detoxification (9), augmenting DNA repair mechanisms (10), activation of epithelial mesenchymal transition (EMT) (11), and evasion of apoptosis (10). These changes disrupt the normal balance within the cells, rendering them more susceptible to proliferation and survival, thus avoiding cell death. Therefore, identifying key role players involved in drug resistance is a viable strategy to tackle this issue, and non-coding RNAs (ncRNAs) are of significant interest.

The human genome is composed of only around 1-2% protein-coding sequences with the remaining ~98% comprising non-protein coding sequences (**Figure 1**) (12). The non-coding segment includes transposons, introns, pseudogenes, and regulatory elements including ncRNAs (13). Transcripts known as ncRNAs, which encompass circular RNAs (circRNAs), long non-coding RNAs (lncRNAs), and microRNAs (miRNAs) are a large group of transcripts that are devoid of protein coding potential. Previously, they were recognized as by-products of transcription that did not serve any biological purpose (14). In recent years, there has been a significant accumulation of evidence suggesting that ncRNAs are crucial regulators in almost all cellular processes, such as transcription, apoptosis, proliferation, and differentiation (15, 16). In addition, it is now well established that impaired non-coding RNA expression, particularly that of lncRNAs, miRNAs and circRNAs is involved in resistance to different cancer therapies (17, 18). Therefore, this review highlights the crucial roles played by these three types of non-coding RNA in the development of drug resistance in cervical cancer (**Figure 2**). In addition, this review also discusses the potential of targeting ncRNAs as a strategy to address treatment resistance in cervical cancer. Previous reviews have largely examined a single ncRNA class or a different biological focus. For example, Zhou et al. (19) reviewed the role of lncRNAs alone in mediating cervical cancer resistance, while Heidari-Ezzati et al. (20) examined lncRNAs and circRNAs jointly but through miRNA-sponging and tumorigenesis rather than chemoresistance. To our knowledge, no review has integrated all three ncRNA classes within a chemoresistance-specific framework, mapped against specific chemotherapeutic agents and targets. These data will assist in early diagnosis and prognostic assessments of cervical cancer. Moreover, it will also contribute to the formulation of non-coding RNA-based treatment strategies for individuals diagnosed with cervical cancer.

**Figure 1:**
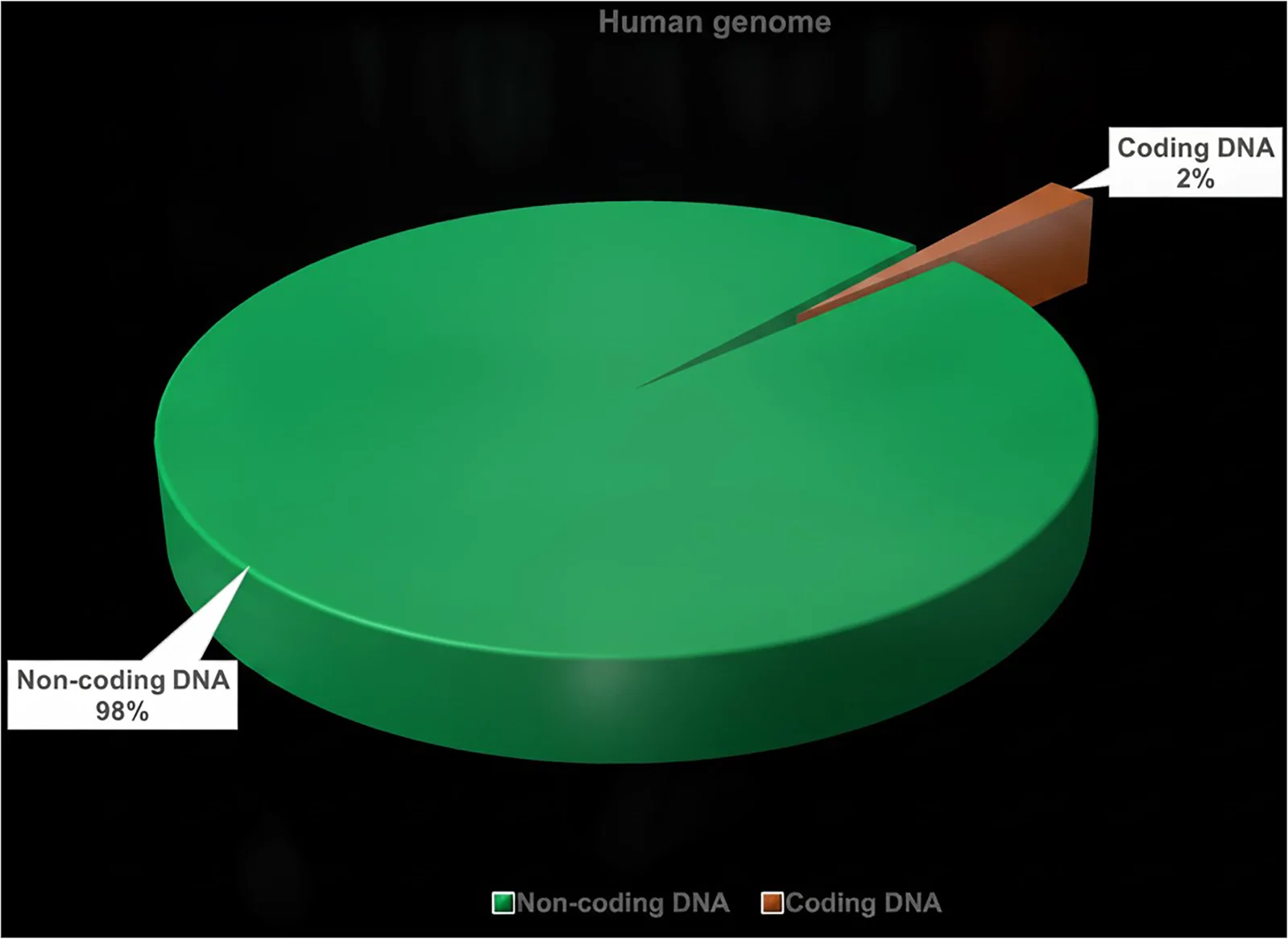
Only around two percent of the human genome consists of protein-coding genes, the other 98% is non-coding DNA. The remaining large chunk (98%) are not to be dismissed as junk; it serves critical roles including the regulation of gene expression.

**Figure 2:**
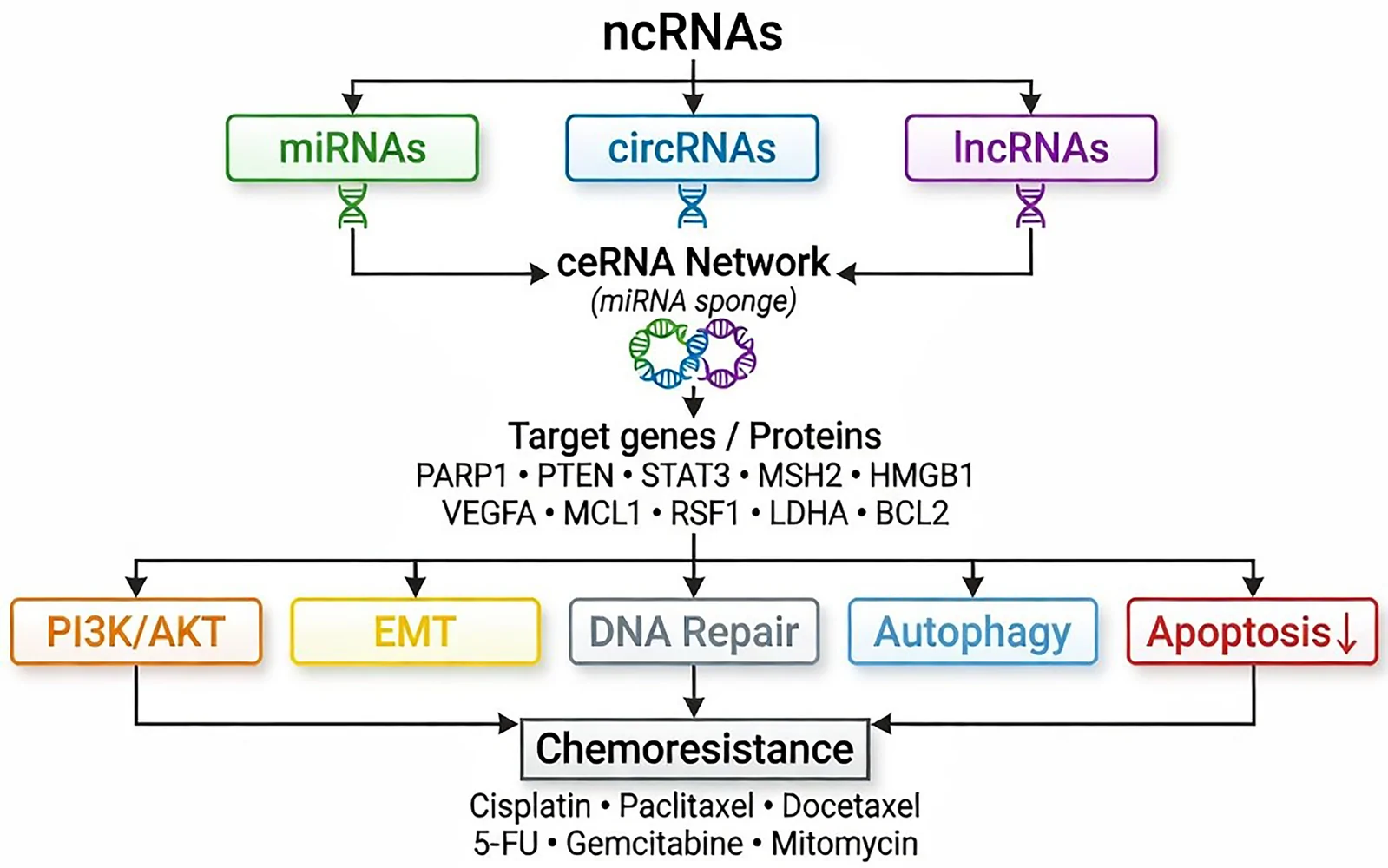
Convergence of non-coding RNA classes during cervical cancer chemoresistance. These ncRNAs act through shared downstream mechanisms, which collectively give rise to clinical chemoresistance against certain therapeutic drugs.

### 1.1 Chemoresistance mediated through miRNAs

MicroRNAs were first discovered in 1993 when a small RNA transcript produced by the lin-4 gene in *Caenorhabditis elegans* was described (21). It was later established that miRs are non-coding ribonucleic acids (RNAs) that vary in length from 18 to 22 bases. They are known to bind to the 3’ untranslated region (UTR) of their target mRNAs (22). Their key function is reported to regulate both transcription and translation (23). MicroRNAs are located in introns or exons (22) and some are found in the chromosomal fragile sites, which indicates that they play a major role in carcinogenesis (24). In addition, cervical cancer drug resistance has also been described as modulated by miRNAs (25).

A variety of chemotherapeutic agents are available for the treatment of cervical cancer, which include platinum-based drugs such as cisplatin (CDDP) and carboplatin, taxanes like paclitaxel, and additional agents, among which is the targeted therapy bevacizumab (26). These chemotherapeutic drugs work mainly by blocking cancer cell growth and spread through DNA damage and replication interference (27, 28). However, research has shown that miRNAs can interfere with these mechanisms, ultimately driving resistance across multiple chemotherapy subtypes (29, 30).

This outcome was first demonstrated by Miller et al. (31), who reported distinct microRNA expression patterns between tamoxifen-sensitive and tamoxifen-resistant MCF-7 breast cancer cells (31). Subsequently, a growing body of research has linked miRNAs to chemotherapy resistance. For example, miR-155 and miR-21 have been identified as contributors to 5-fluorouracil resistance in colorectal cancer cell lines by disrupting the DNA repair mechanisms (32, 33). Moreover, miR-125b, which targets p53, was reported to inhibit the apoptosis that leads to doxorubicin resistance in Ewing sarcoma/primitive neuroectodermal tumors (34). The expression level of this microRNA is increased in doxorubicin-resistant Ewing sarcoma cells when compared to doxorubicin-sensitive cell lines (34).

Like in other malignancies, miRNAs contribute to chemoresistance in cervical cancer (**Figure 3**). The earliest studies about the role of miRNA in cervical cancer drug resistance date back to 2012 when miR-145 was recognized as a component of a p53-miR-145 axis that modulates the IGF signaling pathway, which is implicated in mitomycin resistance (35). Research has been consistently accumulating on this topic. For example, the overexpression of miR-375 has been shown to enhance resistance to paclitaxel in both *in vitro* (SiHa and CaSki) and *in vivo* studies (mice) related to cervical cancer (36, 37). In cervical cancer, the induction of miR-375 through paclitaxel promotes epithelial–mesenchymal transition by directly targeting E-cadherin, thus activating proliferation, and consequently resulting in chemoresistance in cervical cancer cells (36, 37). However, this conflicts with other cervical cancer literature (38). For example, Bierkens et al. (38) have identified hsa-miR-375 as a tumor suppressor gene in cervical carcinogenesis, reporting that its loss correlates with reduced expression, and that ectopically re-expressing it in cervical carcinoma cell lines actually reduced cell viability, directly opposing the effect reported by Shen et al. (36, 37).

**Figure 3:**
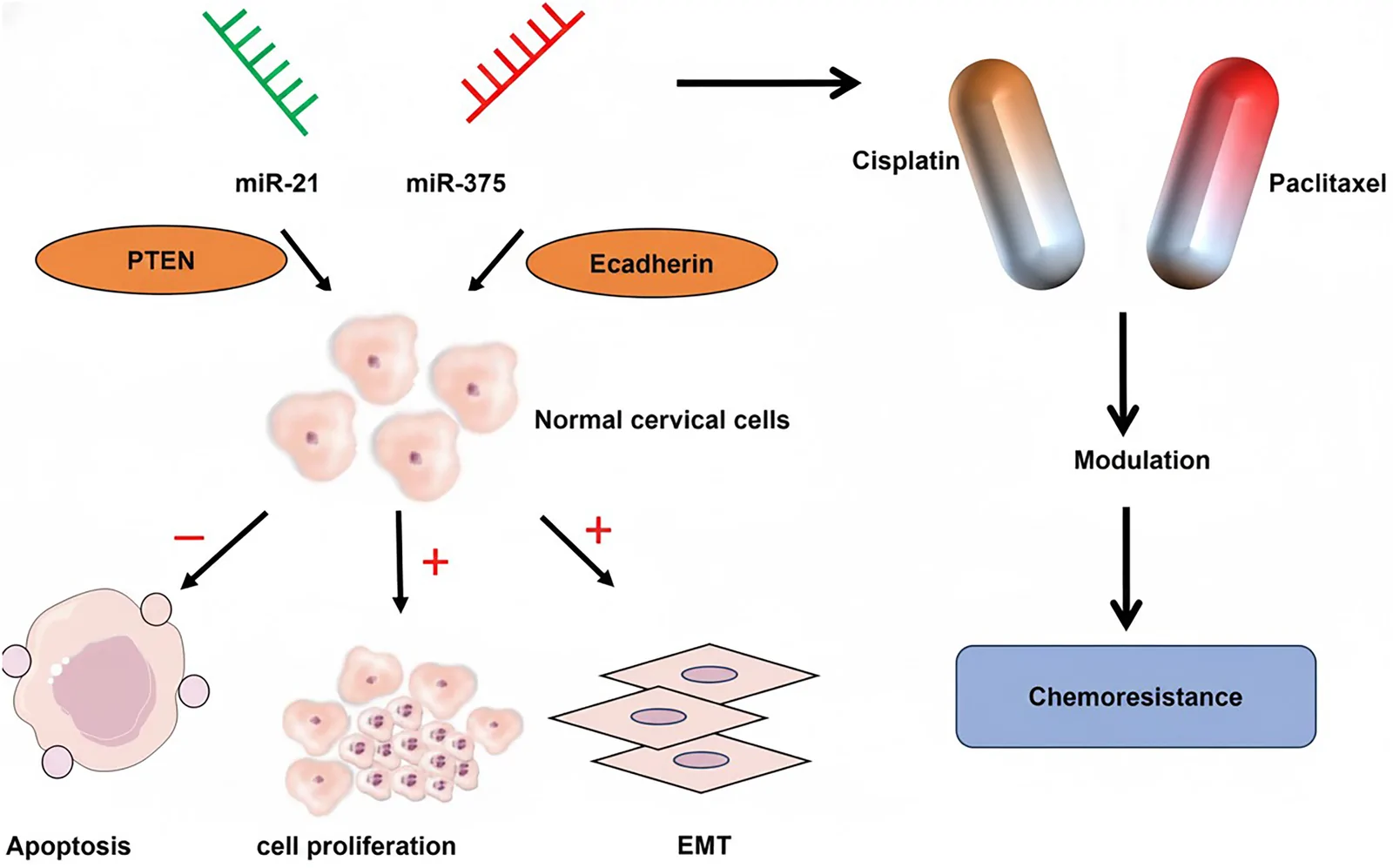
The role of microRNAs in inducing drug resistance in cervical cancer. MicroRNAs target certain proteins such as E-cadherin and PTEN, the combined loss or dysfunction of these proteins promote resistance to chemo drugs.

Beyond initiating carcinogenesis, HPV oncoproteins E6 and E7 also directly regulate specific ncRNAs that later resurface as chemoresistance drivers. Shi et al. (35) provided early evidence of this link by showing that cortisol-induced HPV-E6 expression suppresses p53-dependent miR-145 transcription, and this glucocorticoid-driven E6-p53-miR-145 axis blunts chemotherapy-induced apoptosis in cervical cancer cells, providing an early mechanistic link between HPV oncoprotein signaling and chemoresistance. A more direct connection to the ncRNAs discussed above comes from a study by Wang et al. (39), which showed that HPV16 E7 transactivates lncRNA MALAT1 through promoter binding in complex with SP1, independently of pRb inactivation. Notably, this same MALAT1 later sustains the miR-370-3p/STAT3 feedback loop that drives CDDP resistance (40). Together, these findings indicate that HPV oncoprotein signaling not only initiates carcinogenesis but may also prime the expression of specific ncRNAs that are subsequently co-opted during chemoresistance acquisition, an axis that remains underexplored relative to standard miRNA/mRNA mechanisms.

Returning to individual miRNA-target mechanisms, Yang et al. (41) discovered that CDDP-resistant SiHa and HeLa cervical cancer cells highly express miR-7–5p. The increased expression of miR-7–5p prevented DNA repair by altering the expression of poly (ADP-ribose) polymerase 1 (PARP-1), reduced energy consumption, and promoted autophagy through the suppression of Bcl-2 expression. These findings suggested that miR-7–5p preserved homeostasis during CDDP therapy by promoting energy production while decreasing energy consumption, thus exerting a protective effect on cervical cancer cells exposed to CDDP (41). In addition to the microRNAs noted above, several other microRNAs that are upregulated during drug resistance in cervical cancer are detailed in **Table 1**. Based on this information, clearly microRNA interference or destruction can be a promising therapeutic strategy for cervical cancer cells that exhibit resistance to certain drugs.

**Table 1:** MicroRNAs highly expressed in chemo resistant cervical cancer cells.

MicroRNA	Target	Drug resistance	Model system	Ref
miR-181a	Protein Kinase C delta (PRKCD)	Cisplatin	Clinical (cervical squamous cell carcinoma tissues). Animal (mice). Cell line (SiHa).	42
miR-21	PTEN	Cisplatin	Clinical (cervical cancer tissues). Cell lines (SiHa, Me180, HCE1, HeLa and CaSki).	43
miR-424	Unknown	Paclitaxel	Clinical (cervical squamous cell carcinoma tissues). Cell lines (SiHa and CaSki).	37
miR-27a	Multidrug Resistance 1(MDR1)/P-glycoprotein	Doxorubicin; Vinblastine	Cell line (KB-V1).	44
miR-451	P-glycoprotein	Doxorubicin; Vinblastine	Cell line (KB-V1).	44

### 1.2 circRNAs-mediated chemoresistance

Circular RNAs (circRNAs), which were first identified more than 4 decades ago (45), represent a class of endogenous ncRNAs characterized by circular structure, which is produced from pre-mRNAs through back-splicing (46). This novel class of endogenous ncRNAs is devoid of 5′ caps and 3′ poly (A) tails (46). Due to variations in splicing mechanisms, three main types of circRNAs are generated, including, exon-intron circRNAs (EIciRNAs), exonic circRNAs (ecircRNAs), and circular intronic RNAs (ciRNAs) (47). In comparison to linear RNAs, circRNAs are more stable and demonstrate resistance to RNase R digestion (48). Many circRNAs have been identified as sponges for microRNAs, interact with RNA-binding proteins, modulate gene transcription, and can be translated into proteins (49).

While the functions of most circRNAs remain largely unexplored, some specific circRNAs have been identified as novel players in normal physiological and pathological conditions including cancer (50). A growing body of evidence indicates that circRNAs are linked to hallmarks of cancer, including activation of invasion and metastasis, sustained proliferative signaling and evading growth suppressors (51, 52). In addition, aberrantly expressed circRNAs have been associated with resistance to various cancer treatments, including chemotherapy (53). For instance, in the context of breast cancer cell lines, the overexpression of hsa_circ_0006528 facilitated chemoresistance to adriamycin by sponging miR-7-5p (54). These results highlight the important biological role of circRNAs in chemoresistance experienced by breast cancer patients. In a different study, circSFMBT2 was reported to be upregulated in docetaxel (DTX)-resistant prostate cells and it was demonstrated that this circRNA could augment DTX resistance in prostate tumors *in vivo* through the modulation of the miR-136-5p/TRIB1 axis (55). Moreover, Zang et al. (56) showed that circ-0000337 and JAK2 exhibited elevated expression levels in CDDP-resistant esophageal cancer cells and clinical tumor tissue samples. The highly expressed circ-0000337 derived from exosomes enhanced both cell proliferation and CDDP resistance in esophageal cancer cells when the analysis was done in both *in vitro* and *in vivo* studies (56). Despite the established role of circRNAs in different malignancies that exhibit resistance to certain drugs, there is still much to learn about the functions and mechanisms of circRNAs involved in cervical cancer, especially their roles in drug resistance (**Figure 4**).

**Figure 4:**
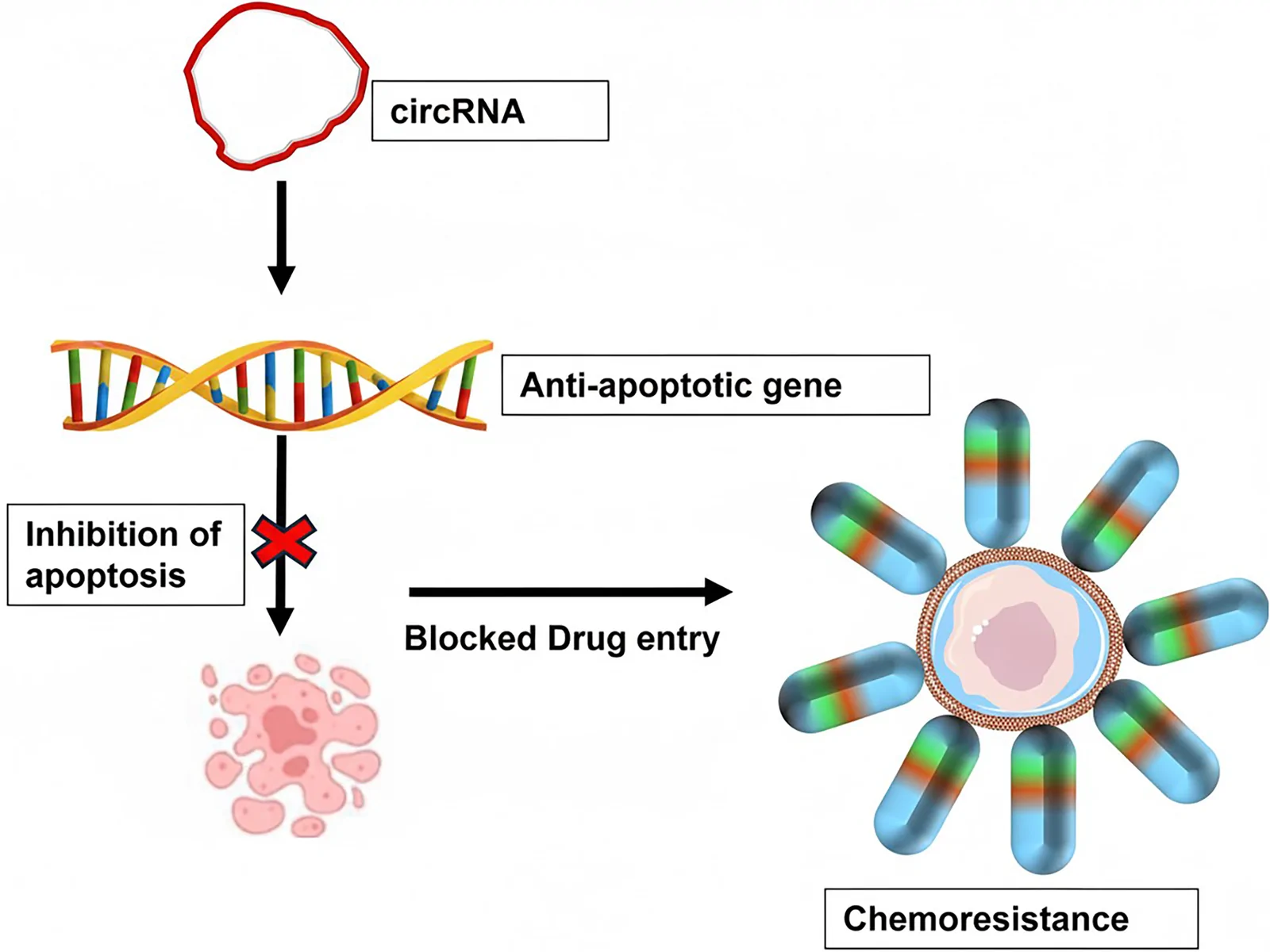
circRNAs are associated with resistance against cervical cancer drugs. They induce drug resistance by various mechanisms, one of which involves functioning as ceRNAs, which leads to the upregulation of anti-apoptotic proteins.

There are several circRNAs that are markedly upregulated in resistant cervical cancer cells, each operating through distinct molecular mechanisms. For example, Guo et al. (57) showed that increased hsa_circ_0023404 expression in cervical cancer cell lines reduced Beclin1 and increased p62, via a miR-5047 sponge effect. This autophagy inhibition resulted in decreased apoptosis and increased CDDP resistance. Moreover, another circRNA, circMTO1, has been identified to upregulate S100A1 expression through sponging miR-6893, which in turn facilitated tumorigenesis and CDDP resistance in cervical cancer cell lines and tumor tissue samples (58). However, it must be noted that circMTO1’s role as a tumorigenic driver in cervical cancer (58) conflicts with its role in other cancer types. For example, circMTO1 was found to suppress hepatocellular carcinoma growth by sponging miR-9 to upregulate p21 (59). This indicates that circMTO1 plays dual, opposing roles depending on which microRNA it sponges and which downstream pathway it affects. In keeping with the pattern of resistance-promoting circRNAs, circ_0004488 has similarly been shown to function as a sponge for miR-136, heightening the expression of Mex-3 RNA-binding family member C (MEX3C), which is a direct target gene of miR-136, ultimately augmenting the resistance of cervical cancer cells to paclitaxel in both *in vitro* and *in vivo* settings (60). In addition, other circRNAs that are implicated in cervical cancer drug resistance are detailed in **Table 2**.

**Table 2:** circRNAs associated with drug resistance in cervical cancer.

circRNAs	Drug resistance	miRNA sponge	Downstream target	Model system	Ref
circPRDM4	Cisplatin	Unknown	USF1/PD-L1 axis	Cell lines (HeLa and SiHa)	61
circ_ZFR	Paclitaxel	miR-944	IL-10	Clinical (cervical squamous cell carcinoma and cervical adenocarcinoma tissues). Cell lines (SiHa and HeLa).	62
circSYT15	Cisplatin	miR-503-5p	RSF1	Clinical (cervical cancer tissues). Animal (mice). Cell lines (SiHa and HeLa).	63
circEPSTI1	Cisplatin	miR-370-3p	MSH2	Clinical (cervical cancer tissues). Cell lines (CaSki, SiHa and HeLa).	64
circ_0001589	Cisplatin	miR-1248	HMGB1	Clinical (cervical cancer tissues). Animal (mice). Cell lines (ME180 and CaSki).	65

### 1.3 Chemoresistance mediated through lncRNAs

LncRNAs are non-coding RNA molecules that are longer than 200 nucleotides (coding potential of < 100 amino acids), characterized by lack of a defined open reading frame (66, 67). Based on genomic location, lncRNAs are classified into five types: antisense lncRNAs transcribed from the antisense strand of protein-coding genes, long intergenic lncRNAs transcribed from DNA sequences between protein-coding genes, long intronic non-coding RNAs transcribed from introns of protein-coding genes, sense lncRNAs transcribed from the sense strand of protein-coding genes and lastly bidirectional lncRNAs transcribed from the opposite strand of the protein-coding genes, in opposite direction and within 1000bp of the promoter of the protein coding genes (68). Long non-coding RNAs have been implicated in carcinogenic processes, including cancer stem cell initiation, cell proliferation, metastasis, inhibition of apoptosis and drug resistance (13, 69–71).

Recent investigations have shown that the expression of lncRNAs is altered in cancers, and these transcripts are involved in numerous aspects of drug resistance (72–74). For example, the overexpression of PVT1 lncRNA is associated with CDDP-resistance in gastric cancer cell lines and clinical tumor tissue samples due to the inhibition of apoptosis (75). Additionally, Li et al. (76) reported that a different lncRNA, known as HOTAIR, was highly expressed in ovarian cancer, leading to the activation of the Wnt/β-catenin signaling pathway and enhanced cell cycle progression, which was associated with induction of CDDP resistance in ovarian cancer cell lines and clinical tumor tissues. In another study, it was established that lncRNA HULC elevated drug resistance of hepatocellular carcinoma cell lines and tumor tissues to oxaliplatin, 5-FU, and pirarubicin by initiating autophagy via stabilization of Sirtuin 1 (SIRT1) (77).

Mirroring findings from other cancer types, lncRNAs are also implicated in cervical cancer drug resistance (**Figure 5**) (19, 78). To illustrate, the expression of UCA1 is significantly increased in CDDP-resistant cervical cancer cells. The results also showed that UCA1 inhibited apoptosis by downregulating caspase 3 and upregulating CDK2, while also promoting cell proliferation by survivin levels and decreasing p21 levels (79). Similarly, lncRNA HOTAIR enhanced CDDP, paclitaxel and docetaxel chemoresistance in cervical cancer by inducing EMT and modulating the miR-29b/PTEN/PI3K pathway under *in vitro* conditions (80). In another investigation, Shen et al. (81) revealed that lncRNA PVT1 acted as a sponge for miR-195 and regulated EMT in cervical cancer cell lines, leading to modulation of paclitaxel chemoresistance. Additional examples of lncRNAs involved in the regulation of chemoresistance in cervical cancer are detailed in **Table 3**. Targeting these lncRNAs could reverse drug resistance in cervical cancer.

**Figure 5:**
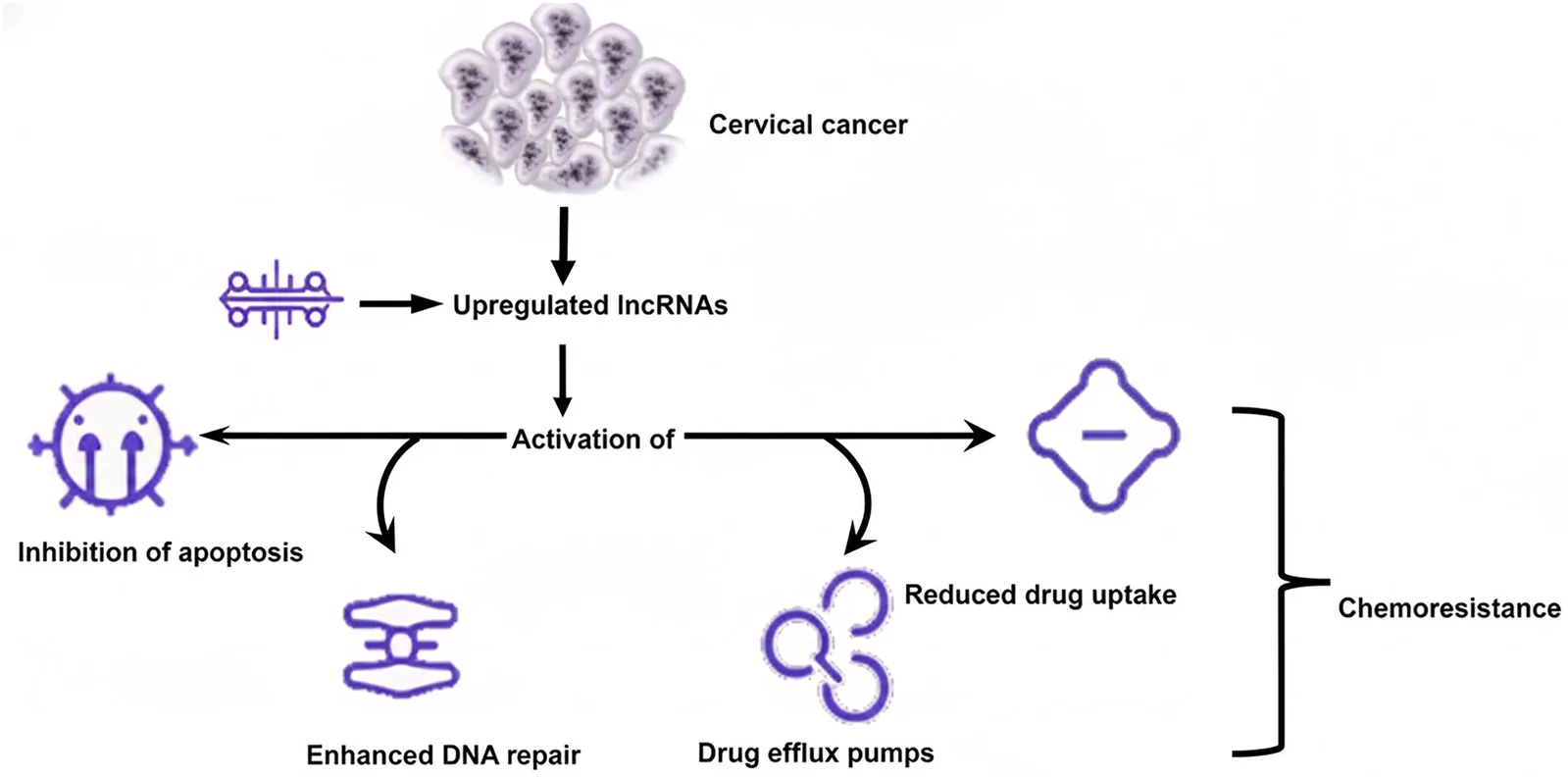
Chemoresistance mediated through lncRNAs in cervical cancer. LncRNAs induce chemoresistance by activating various intricate mechanisms, such as enhanced DNA repair, the operation of drug efflux pumps, inhibition of apoptosis, and reduced drug intake.

**Table 3:** LncRNAs deregulated during cervical cancer chemoresistance.

LncRNA	Chemotherapy agent(s)	Key target/pathway	Model system	Ref
MALAT1	Cisplatin	Wnt/β-catenin	Cell lines (HeLa and C-33A).	82
NEAT1	5-fluorouracil	miR-34a, LDHA	Clinical (cervical cancer tissues). Cell lines (CaSki, HeLa, SiHa and C-33A).	83
NCK1-AS1	Cisplatin	miR-134-5p, MSH2	Clinical (cervical cancer tissues). Cell line (HeLa).	84
LINC00460	Cisplatin	TGFBI	Clinical (cervical cancer tissues). Animal (mice). Cell lines (SiHa, Me180, HeLa and CaSki).	85
DLG1-AS1	Gemcitabine	miR-16-5p, HDGF	Animal (mice). Cell lines (HeLa and SiHa).	86
linc00958	Cisplatin	AKT1/GSK3β/VEGFA pathway	Animal (mice). Cell line (SiHa).	87
ZFAS1	Cisplatin	SP1	Clinical (cervical cancer tissues). Animal (mice). Cell lines (CaSki, C-33A and HeLa).	88
SNHG12	Cisplatin	WEE1, miR-503-5p	Clinical (cervical cancer tissues). Animal (mice). Cell lines (HeLa and CaSki).	89

### 1.4 ceRNA networks and crosstalk between ncRNA classes in cervical cancer chemoresistance

The individual mechanisms of ncRNAs outlined above do not act in isolation; miRNAs, lncRNAs and circRNAs are proposed to communicate through a shared regulatory code operating at the post-transcriptional level. According to the competing endogenous RNA (ceRNA) hypothesis, mRNAs, pseudogenes, lncRNAs and circRNAs can all carry microRNA response elements (MREs), placing them in a direct competition for a common pool of miRNAs (90). When a lncRNA or circRNA binds and sequesters a specific miRNA, it de-represses miRNA’s other mRNA targets, effectively linking unrelated transcripts into a single regulatory network (90). This framework explains why isolated lncRNA-, circRNA- and miRNA-driven resistance mechanisms recur across independent studies of cervical cancer, and why treatments targeting one ncRNA class often fail to fully reverse chemoresistance.

Direct experimental support for such multi-class crosstalk in cervical cancer chemoresistance comes from a whole-transcriptome profiling of CDDP-resistant SiHa cells (91). To compare parental SiHa cells with CDDP-resistant SiHa cells, Lv et al. (91) constructed parallel lncRNA-miRNA-mRNA and circRNA-miRNA-mRNA ceRNA networks from the same resistant phenotype and went on to identify and experimentally validate lncRNA-AC010198.2/miR-34b-3p/STC2 as a functional axis driving CDDP resistance. This work demonstrates that lncRNAs and circRNAs are not independent contributors to resistance but can converge on overlapping miRNA pools within the same resistant cell population, supporting a model of an interconnected, rather than a compartmentalized, regulatory landscape.

Revisiting the axes discussed earlier reveals a similar pattern of convergence. LncRNA NEAT1 acts as a ceRNA by sponging miR-34a to de-repress LDHA, sustaining glycolysis and 5-fluorouracil resistance in cervical cancer cells (83). The same oncogenic dependency on NEAT1 is what makes it an attractive RNAi/shRNA target elsewhere in this review. Comparable regulatory logic is observed for circMTO1, which sponges miR-6893 to upregulate S100A1 and confers CDDP resistance (58); for lncRNA HOTAIR, which sponges miR-29b to modulate PTEN/PI3K signaling and EMT-associated chemoresistance (80); and for circ_0004488, which sponges miR-136 to upregulate MEX3C and promote paclitaxel resistance (60). Although the direct physical crosstalk between these particular lncRNA and circRNA axes has not yet been formally tested in cervical cancer, their shared sponge-and-derepress logic, converging on overlapping pathways such as PI3K/AKT signaling and apoptosis regulation, illustrates how independently discovered ncRNAs may in fact belong to a shared regulatory network rather than acting through unrelated mechanisms.

### 1.5 Mechanistic classification of ncRNAs by biological process in cervical cancer chemoresistance

The ncRNAs discussed so far are currently grouped by transcript class (miRNA, circRNA, lncRNA). However, what drives chemoresistance is the biological processes they converge on. These processes include augmented DNA repair, EMT, evasion of apoptosis, dysregulated autophagy, and enhanced drug efflux. Several of the ncRNAs already discussed above act on the same biological process (**Table 4**) despite belonging to different RNA classes. Therefore, this section groups the ncRNAs already presented above by biological process rather than RNA type, making these cross-class mechanistic convergences explicit.

**Table 4:** ncRNAs grouped by the biological process they mediate during cervical cancer chemoresistance.

Mechanism	ncRNA (class)	Target/pathway	Drug(s)	Ref
DNA repair	SNHG12 (lncRNA)	miR-503-5p/WEE1	Cisplatin	89
DNA repair	NCK1-AS1 (lncRNA)	miR-134-5p/MSH2	Cisplatin	84
DNA repair	circEPSTI1 (circRNA)	miR-370-3p/MSH2	Cisplatin	64
DNA repair	miR-7-5p (miRNA)	PARP-1	Cisplatin	41
EMT	HOTAIR (lncRNA)	miR-29b/PTEN/PI3K	Cisplatin, paclitaxel, docetaxel	80
EMT	miR-375 (miRNA)	E-cadherin	Paclitaxel	36, 37
EMT	circ_0001589 (circRNA)	miR-1248/HMGB1	Cisplatin	65
EMT	PVT1 (lncRNA)	miR-195 sponge	Paclitaxel	81
Apoptosis evasion	miR-320a (miRNA)	MCL1	Cisplatin	92
Apoptosis evasion	UCA1 (lncRNA)	Caspase 3↓, CDK2↑, survivin↑, p21↓	Cisplatin	79
Apoptosis evasion/Autophagy	hsa_circ_0023404 (circRNA)	miR-5047/Beclin1/p62	Cisplatin	57
Drug efflux	miR-27a (miRNA)	MDR1/P-glycoprotein	Doxorubicin, vinblastine	44
Drug efflux	miR-451 (miRNA)	P-glycoprotein	Doxorubicin, vinblastine	44

### 1.6 ncRNA-based delivery strategies to overcome chemoresistance in cervical cancer

Multiple delivery strategies are being explored for ncRNA-based therapeutics to overcome chemoresistance in cervical cancer. Engineered exosome-mediated delivery represents one such approach. Zhou et al. (92) demonstrated that miR-320a is downregulated in CDDP-resistant cervical cancer tissue and cell lines (HeLa and CaSki). The authors engineered exosomes to package and deliver miR-320a into these resistant cells, restoring miR-320a levels and thereby suppressing MCL1, an anti-apoptotic protein that miR-320a directly targets. This effect resensitized the cells to CDDP both *in vitro* and in a mouse xenograft model, demonstrating that exosome-mediated delivery is effective in reversing acquired chemoresistance.

Beyond exosome-mediated delivery, RNAi/siRNA-based approach also offers considerable promise. As reviewed by He et al. (93), siRNAs and shRNAs can be used to silence chemoresistance-driving lncRNAs, such as NEAT1, through the RISC pathway. Targeting these oncogenic lncRNAs suppresses proliferation, migration, and chemoresistance in cervical cancer models. In addition, the nanostructure-based co-delivery of ncRNA and chemotherapeutics is another strategy worth highlighting. A review by Xie and Xu (94) discussed stimuli-responsive nanoparticles designed to co-deliver siRNA alongside chemotherapy drugs or natural compounds directly to cervical tumor tissue. This approach improved local drug accumulation and allowed controlled release at the tumor site, ultimately enhancing anticancer activity while helping to prevent and reverse drug resistance.

### 1.7 Towards clinical application: ncRNA biomarkers, liquid biopsy, and RNA-based therapeutics in cervical cancer

#### 1.7.1 Predictive biomarkers of chemotherapy response

A growing body of evidence supports ncRNAs as predictors of chemotherapy response in cervical cancer, particularly for neoadjuvant chemotherapy (NACT) and concurrent chemoradiotherapy (CRT) in locally advanced diseases (95). In a study of paired pre- and post-treatment plasma samples from patients with locally advanced cervical cancer, circulating miR-326 was identified as a candidate biomarker capable of distinguishing NACT responders from non-responders with useful diagnostic accuracy (95). Similarly, plasma profiling before and after CRT showed specific post-treatment reductions in hsa-miR-34a-5p, hsa-miR-34c-5p, and hsa-miR-409-3p, while other measured miRNAs remained stable, supporting their candidacy as response-monitoring biomarkers (96).

However, it is important to note that predicting the degree of tumor response to chemotherapy or radiotherapy remains the central unmet clinical need in locally advanced cervical cancer. This is largely because there is no single miRNA with the consistency required for clinical translation, pointing to combined multi-marker panels, rather than single miRNAs, as the more realistic path forward (97).

#### 1.7.2 Liquid biopsy applications: circulating miRNAs and exosomal ncRNAs

Liquid biopsy approaches rely on the fact that ncRNAs are protected from plasma ribonucleases when packaged within extracellular vesicles or ribonucleoprotein complexes, a property that allows for reproducible and minimally invasive serial sampling over the course of disease and treatment (98). In cervical cancer specifically, exosomal circSLC26A4 has been investigated as a diagnostic liquid biopsy marker, with plasma exosomal circSLC26A4 levels significantly elevated in cervical cancer patients compared with samples from healthy women and positively correlating with both lymph node metastasis and FIGO stage (99).

More broadly, exosomes are now understood as active regulators of intercellular communication rather than transcriptional by-products, packaging miRNAs, lncRNAs, and circRNAs depending on cancer type. This dual role of serving simultaneously as passive biomarkers and active mediators of drug-resistance in tumour cells has been documented across different cancer types, including cervical cancer (100). The findings in cervical cancer showed that exosomal ncRNAs contribute mechanistically to chemotherapy and radiotherapy resistance while simultaneously showing promise as liquid biopsy markers capable of tracking resistance non-invasively (100). This liquid-biopsy role, however, does not fully capture the mechanistic functions of exosomal ncRNAs.

Accumulating evidence indicates that exosomes are active agents of horizontal transfer of chemoresistance between tumor cells. In cervical cancer specifically, Chen et al. (63) demonstrated that CDDP-resistant cells secrete circSYT15-enriched exosomes that, once internalized by recipient cells, drive CDDP resistance via the miR-503-5p/RSF1 axis, directly demonstrating exosome-mediated resistance transfer between cervical cancer cells. Hu et al. (40) reported a related feedback mechanism involving exosomal lncRNA MALAT1 in cervical cancer, where a MALAT1/miR-370-3p/STAT3 loop, sustained through exosomal transfer, activates PI3K/Akt signaling and mediates CDDP resistance. These findings suggest that exosomal ncRNA exchange between cells is a recurring theme of cervical cancer biology rather than an isolated observation (40, 63). A comparable mechanism has been reported in esophageal cancer, where resistant cells release exosomal circ-0000337 that confers CDDP resistance on recipient cells through the miR-377-3p/JAK2 axis (56). Taken together, these findings position exosomal ncRNAs not as merely passive markers of an existing resistant state but as active mediators capable of converting drug-sensitive cells into a resistant phenotype.

Beyond driving horizontal transfer of chemoresistance between tumor cells, ncRNA also shapes the tumor immune microenvironment, a dimension that warrants attention given the growing use of immune checkpoint inhibitors in cervical cancer. USF1-induced circPRDM4 promotes immune escape in CDDP-resistant cervical cancer cells; its knockdown restores peripheral blood mononuclear cell cytotoxic activity and lowers immunosuppressive cytokine output, directly linking chemoresistance-associated circRNA to immune evasion (61). Similarly, circ_ZFR contributes to paclitaxel resistance in cervical cancer partly through miR-944-mediated upregulation of IL-10, an immunosuppressive cytokine known to blunt anti-tumor immune responses (62). Together, these findings suggest that chemoresistance-associated ncRNAs may simultaneously modulate the immune checkpoint landscape, making them potential biomarkers or targets for immunotherapy-chemotherapy strategies in cervical cancer.

#### 1.7.3 Ongoing RNA-based therapeutic strategies

Non-coding RNAs are not only biomarkers of chemoresistance but also emerging as therapeutic targets. A 2024 review of miRNA drug development reported that the first anti-miRNA drugs, including Miravirsen and Cobomarsen, have successfully completed phase II clinical trials, confirming that miRNA silencing is clinically achievable mainly in non-oncological or hematological settings (101). The same review points to MRX34, a nanoparticle-delivered mimic of the tumor-suppressor miR-34a, tested in several solid cancers including cervical cancer, as a relevant model for restoring miRNAs lost during chemoresistance acquisition (101). However, the delivery and immunogenicity issues have kept such agents in early trials (102).

For cervical cancer specifically, a 2025 review describes siRNA and mRNA-based strategies for silencing the HPV E6 and E7 oncogenes using nanoparticle or PEGylated liposome delivery (103). This offers a precision alternative to standard treatment, alongside CRISPR/Cas9 and antisense approaches, but the issue of costs and scalable delivery remain unresolved (103). In addition, exosomes are also being explored as delivery vehicles for siRNAs and antisense constructs aimed at reversing chemoresistance in gynecological cancers, including cervical cancer (100).

### 1.8 Conclusion and future directions

In summary, the dysregulation of ncRNAs plays a critical role in the established mechanisms of drug resistance in cervical cancer. However, the role of other ncRNAs such as piRNAs, tRNA-derived fragments, and snoRNAs in mediating chemoresistance in cervical cancer remain largely unexplored and warrant further investigation. In addition, despite the mechanistic rationale for targeting ncRNAs to reverse chemoresistance in cervical cancer, several translational barriers continue to limit clinical progress. RNA molecules suffer from poor intrinsic stability and a tendency to trigger off-target effects and innate immune activation, constraining dosing and specificity (104). In cervical cancer specifically, achieving tumor-specific delivery remains a key obstacle; engineered exosomes and nanoparticles have been proposed to improve targeting and reduce off-target toxicity, but robust clinical validation is still lacking (93). Another challenge is the limited availability of physiologically relevant, drug-resistant cervical cancer models. Collectively, these barriers: stability, specificity/off-target effects, immunogenicity, lack of physiologically relevant models and delivery efficiency explains why ncRNA-targeted chemoresistance strategies in cervical cancer remain largely preclinical, despite their mechanistic promise. It is also important to note that much of the available evidence especially the ones cited in this study is based on cell-line studies or cohorts with small sample sizes, and this constrains the extent to which these findings can be generalized to broader patient populations.

Addressing these translational gaps will require the adoption of emerging technological platforms. Single-cell sequencing and spatial transcriptomics could resolve ncRNA-mediated chemoresistance within the heterogeneous tumor microenvironment, while multi-omics integration and AI-driven analytical approaches can accelerate the identification of clinically actionable ncRNA biomarkers from complex datasets. From a therapeutic standpoint, CRISPR-mediated ncRNA targeting and nanoparticle-based delivery systems present viable strategies for improving specificity and addressing the stability challenges outlined above. Together, these tools will be essential for advancing ncRNA-based strategies toward clinically validated interventions for chemoresistance in cervical cancer.
